# Prognostic value of neutrophil-to-lymphocyte ratio and platelet-to-lymphocyte ratio in intensity modulated radiation therapy for nasopharyngeal carcinoma

**DOI:** 10.18632/oncotarget.24173

**Published:** 2018-01-11

**Authors:** Yanming Jiang, Song Qu, Xinbin Pan, Shiting Huang, Xiaodong Zhu

**Affiliations:** ^1^ Department of Radiation Oncology, Affiliated Tumor Hospital of Guangxi Medical University, Cancer Institute of Guangxi Zhuang Autonomous Region, Nanning, Guangxi, China

**Keywords:** nasopharyngeal carcinoma, NLR, PLR, prognosis

## Abstract

**Background:**

Inflammatory response markers plays an important role in tumor progression. The aim of this analysis was to evaluate whether the neutrophil-to-lymphocyte ratio (NLR), and platelet-to-lymphocyte ratio (PLR) could predict the prognosis of nasopharyngeal carcinoma(NPC).

**Materials and Methods:**

247 patients who underwent Intensity Modulated Radiation Therapy( IMRT )were enrolled from January 2012 and December 2012. NLR, and PLR were calculated from peripheral blood cell counts taken at pre-treatment. Optimal cutoff values of NLR and PLR were determined on the basis of receiver operating characteristic curve analysis. Overall survival (OS), progression-free survival(PFS), distant metastasis-free survival (DMFS) and loco-regional recurrence-free survival ( LRFS) rates were compared according to NLR and PLR level respectively. Multivariate analysis was performed to assess the prognostic value of NLR and PLR.

**Results:**

The 5-year estimated OS, PFS, LRFS and DFS were 87.2, 77.8, 96.9, and 86.2%, respectively. Our results shows that the NLR was significantly associated with T-stage (*P* < 0.05), N-stage (*P* < 0.05) and tumor stage(*P* < 0.05). PLR was significantly associated with T-stage (*P* < 0.05) and tumor stage(*P* < 0.05). NLR was an independent prognostic indicator for OS (HR: 3.259, *P* = 0.004), PFS (HR:7.093, *P* < 0.001), DMFS (HR: 6.576, *P =* 0.003), except for PLR. In subgroup analysis, adjuvant chemotherapy had no significantly improved survival for patients with high NLR.

**Conclusions:**

NLR is a strong prognostic factor for NPC patients. NLR might not be a useful indicator for selection of treatment strategies for loco-regionally advanced NPC.

## INTRODUCTION

Nasopharyngeal carcinoma (NPC) is a common tumor in southern China and southeast Asia, with a incidence of 20–30/100 000/year in some areas of southern China [1]. The anatomic location of NPC is very complicated, but it is sensitive to radiotherapy, which leads to a favorable prognosis. The cure rate has been significantly improved owing to the use of intensity modulated radiotherapy (IMRT), which led to a significant improved in the local recurrence-free survival and overall survival [2]. However, distant control remains unsatisfactory, distant failure remains a challenge [3]. Therefore, development of novel prognostic indicators is important for NPC treatment.

The tumor, Node, Metastasis (TNM) staging system is widely regarded as the most valuable prognostic factor affecting the treatment of NPC. Recently, more and more evidence confirmed that systemic inflammatory response has been reported to be an independent prognostic biomarker in many types of tumors [4, 5]. Existing researches have shown a significant link between inflammatory markers, such as: neutrophil-to-lymphocyte ratio (NLR), or platelet-to-lymphocyte ratio (PLR), and poor prognosis in several types of tumors [6, 7]. However, the influence of NLR and PLR on the prognosis of NPC under IMRT is not clear. So, the purpose of this research was to evaluate the effect of NLR and PLR on survival in NPC received IMRT.

## RESULTS

### Patient characteristics

Of 247 patients with NPC, the median age was 46 years (range 18–86 years), 197 (79.8%) were male, 50 (20.2%) were female (Table 1). The median values of the pre-treatment neutrophil, lymphocyte and platelet counts were 4.27 ×10^9^/L, 1.86 × 10^9^/L and 242 × 10^9^/L, respectively. The median values of NLR and PLR were 2.28 ( range, 1.75--2.89) and 126.42 ( range, 102.5–169.2), respectively.

**Table 1:** Characteristics of the study population of NPC individuals

Variables	Cases (%)
Age(years)	
> 50/≤ 50	73 (29.6%)/174 (70.45%)
Gender	
Male/Female	197 (79.8%)/50 (20.2%)
T stage	
T1/T2/T3/T4	10 (4%)/62 (25.1%)/79 (32%)/96 (38.9%)
N stage	
N0/N1/N2/N3	11 (4.5%)/76 (30.8%)/134 (54.3%)/26 (10.5%)
Tumor Stage	
I/II/III/IV	1 (0.4%)/23 (9.3%)/117 (47.4%)/106 (42.9%)
Concurrent chemotherapy	
Yes/No	225 (91.1%)/22 (8.9%)
Adjuvant chemotherapy	
Yes/No	119 (48.2%)/128 (51.8%)

### Survival outcomes

Median follow-up time was 53 months (3–64 months). Until the last follow-up, there were 31 patients with death, 54 with progression, 7 patients with local or regional recurrence, and 32 with distant etastasis.The 1-year estimated OS, PFS, LRFS and DFS were 97.5, 94.7, 100, and 96.7%, respectively. The 3-year estimated OS, PFS, LRFS and DFS were 89.4, 81.2, 96.9, and 89.1%, respectively. The 5-year estimated OS, PFS, LRFS and DFS were 87.2, 77.8, 96.9, and 86.2%, respectively.

### The cutoff values for NLR and PLR

The optimum cut-point values of the preoperative NLR and PLR for survival prediction were determined through receiver operating characteristic curve (ROC) curve analysis. When overall survival (OS) was used to an end point for NLR and PLR, the areas under the curve (AUC) for NLR and PLR were 0.720 (*P <* 0.001), 0.579 (*P =* 0.148), respectively . The cutoff values of NLR was 2.73 (sensitivity, 67.74%; specificity, 72.96%). Subsequently, PLR was excluded because of the small AUC (*P >* 0.05). When using progression-free survival (PFS) as an end point, the cut-off values were 2.19 (sensitivity, 87.50%; specificity, 51.76%, *P <* 0.001), 108.33 (sensitivity, 85.42%; specificity, 36.18%, *P =* 0.013), respectively. The NLR and PLR cutoff values for distant metastasis-free survival (DMFS) were 2.20 (sensitivity, 87.10%; specificity, 49.10%, *P <* 0.001) and 137.36 (sensitivity, 64.5%; specificity, 61.1%, *P =* 0.026). When loco-regional recurrence-free survival (LRFS) as an end point for NLR and PLR, the AUC for NLR and PLR were 0.648 (*P <* 0.001), 0.603 (*P =* 0.213), respectively. The cut-off values of NLR was 2.23. All of the cases were divided into two groups, the high groups greater than or equal to the cut-off values,the low groups less than the cutoff values.

Correlation of NLR, PLR with clinical characteristics Table 2 shows the correlation between NLR, PLR and clinical baseline data. Our results shows that the NLR was significantly associated with *T*-stage, *N*-stage and tumor stage (*P <* 0.05), PLR was significantly associated with gender, *T*-stage and tumor stage (*P <* 0.05).

**Table 2:** Correlation of NLR, PLR with clinical characteristics

varibrila	NLR (median, range)	*P*	PLR (median, range)	*P*
Age		0.506		0.477
≤ 50	2.32 (1.67–2.82)		128.95 (103.07–169.97)	
> 50	2.22 (1.82–3.08)		121.67 (98.44–167.24)	
Gender		0.143		0.041
man	2.33 (1.76–2.89)		123.54 (101.36–163.66)	
female	2.11 (1.62–2.72)		149.44 (111.57–188.95)	
T stage		0.002		0.005
T1	1.86 (1.41–2.35)		109.57 (96.33–133.54)	
T2	2.11 (1.52–2.69)		114.68 (95.05–153.08)	
T3	2.36 (1.86–2.90)		121.72 (103.09–185.25)	
T4	2.45 (1.95–3.24)		142.93 (105.85–181.82)	
N stage		0.041		0.157
N0	3.35 (2.16–6.92)		175.61 (111.60–462.96)	
N1	2.15 (1.59–2.76)		116.26 (94.75–164.17)	
N2	2.29 (1.74–2.89)		129.54 (103.16–169.41)	
N3	2.46 (2.08–2.93)		123.24 (109.69–164.13)	
Tumor stage		0.009		0.018
I	2.36 (2.36–2.36)		175.61 (175.61–175.61)	
II	1.84 (1.28–2.55)		114.29 (84.36–133.11)	
III	2.18 (1.70–2.75)		121.68 (100.12–166.91)	
IV	2.50 (1.97–3.23)		140.00 (105.55–180.85)	

NLR, neutrophil count to lymphocyte count; PLR, platelet count to lymphocyte count.

The relationship between clinical prognosis and baseline characteristic Kaplan-Meier analysis was used to calculate patients’ survival. Log-rank test was used to compare the survival rates among different groups.

To determine the effect of NLR and PLR on the survival prognosis, we first performed univariate analysis on the clinical characteristics for prognosis.

The results of univariate analysis for OS, PFS, DMFS and LRFS were showed in Table 3, of the available clinical variables, age (*P =* 0.000),

**Table 3:** Univariate analysis of factors associated with OS, PFS, DMFS and LRFS

variable	OS		PFS		DMFS		LRFS	
	Univariate		Univariate		Univariate		Univariate	
	X^2^	*P*	X^2^	*P*	X^2^	*P*	X^2^	*P*
Age	21.871	0.000	4.205	0.004	0.051	0.822	0.593	0.441
Gender	2.218	0.136	2.795	0.095	1.224	0.269	0.179	0.673
T stage	8.121	0.044	11.617	0.009	11.505	0.009	1.797	0.616
N stage	1.473	0.689	2.665	0.446	7.158	0.065	3.853	0.278
Tumor stage	11.117	0.011	15.103	0.002	19.894	0.000	0.899	0.826
NLR	19.152	0.000	30.875	0.000	18.131	0.000	6.845	0.009
PLR	N/A	N/A	7.882	0.005	5.194	0.023	N/A	N/A

N/A, Not Available.

**Figure 1:**
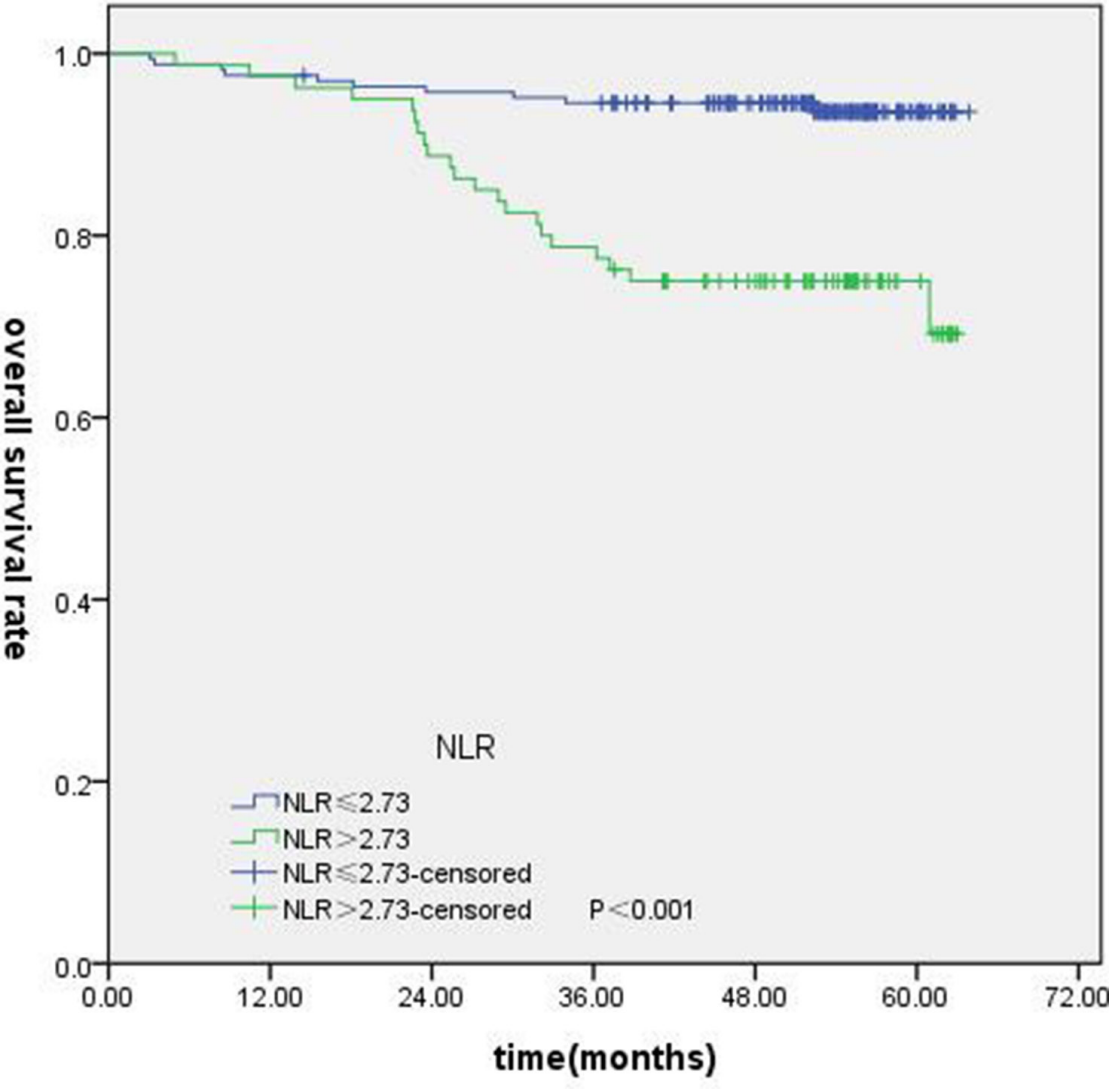
Kaplan-Merier curves for OS according to NLR

**Figure 2:**
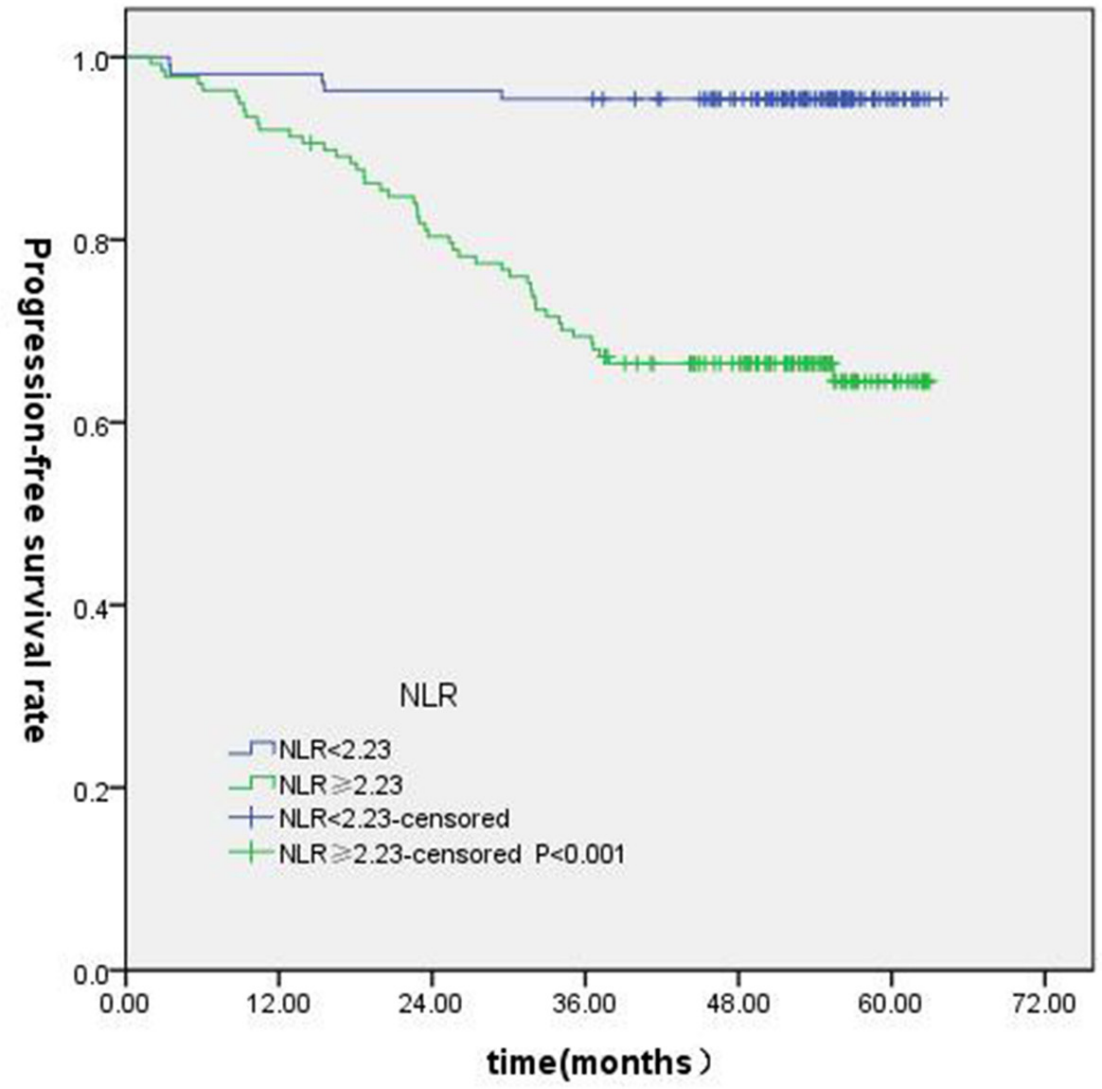
Kaplan-Merier curves for PFS according to NLR

**Figure 3:**
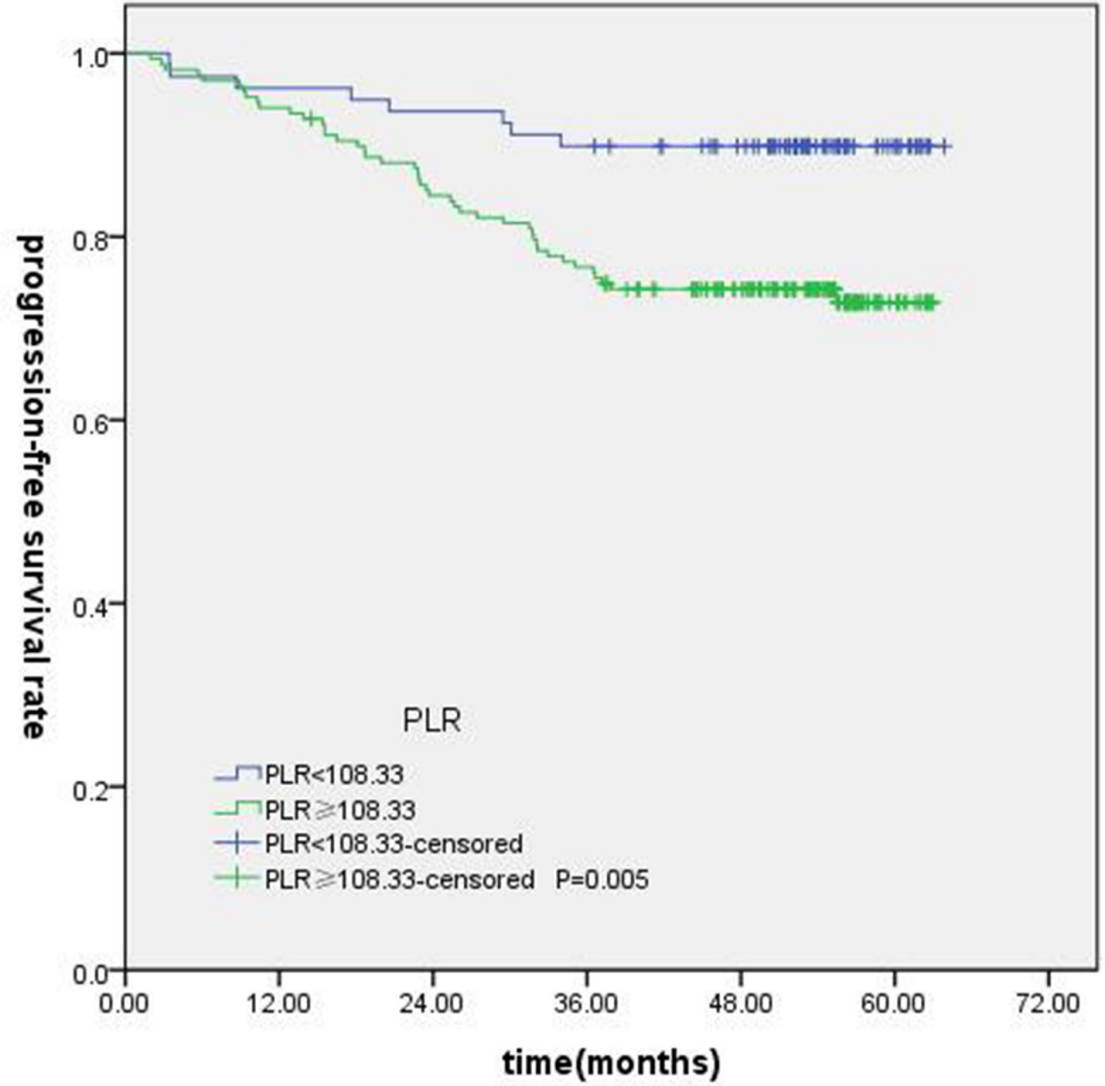
Kaplan-Merier curves for PFS according to PLR

**Figure 4:**
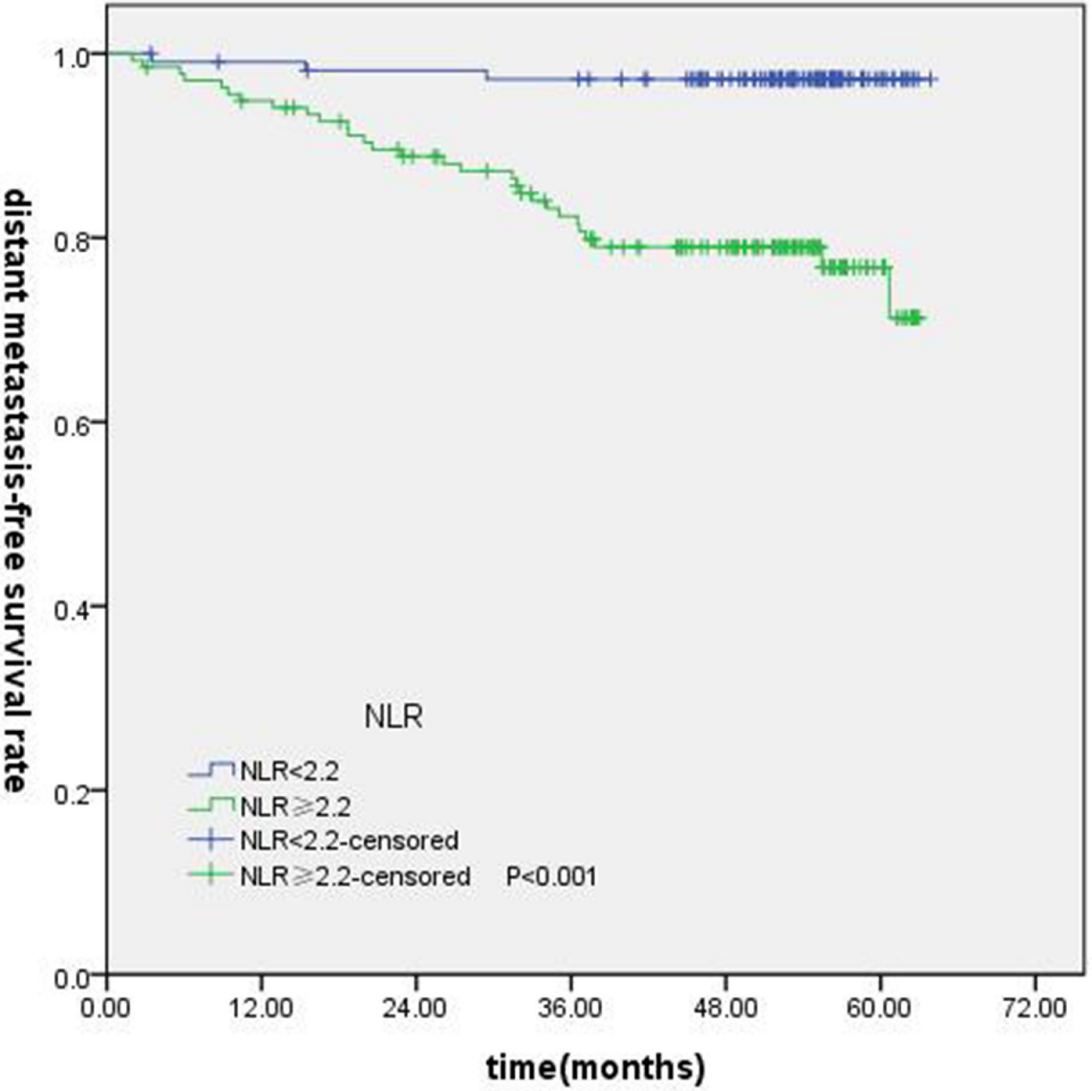
Kaplan-Merier curves for DFS according to NLR

**Figure 5:**
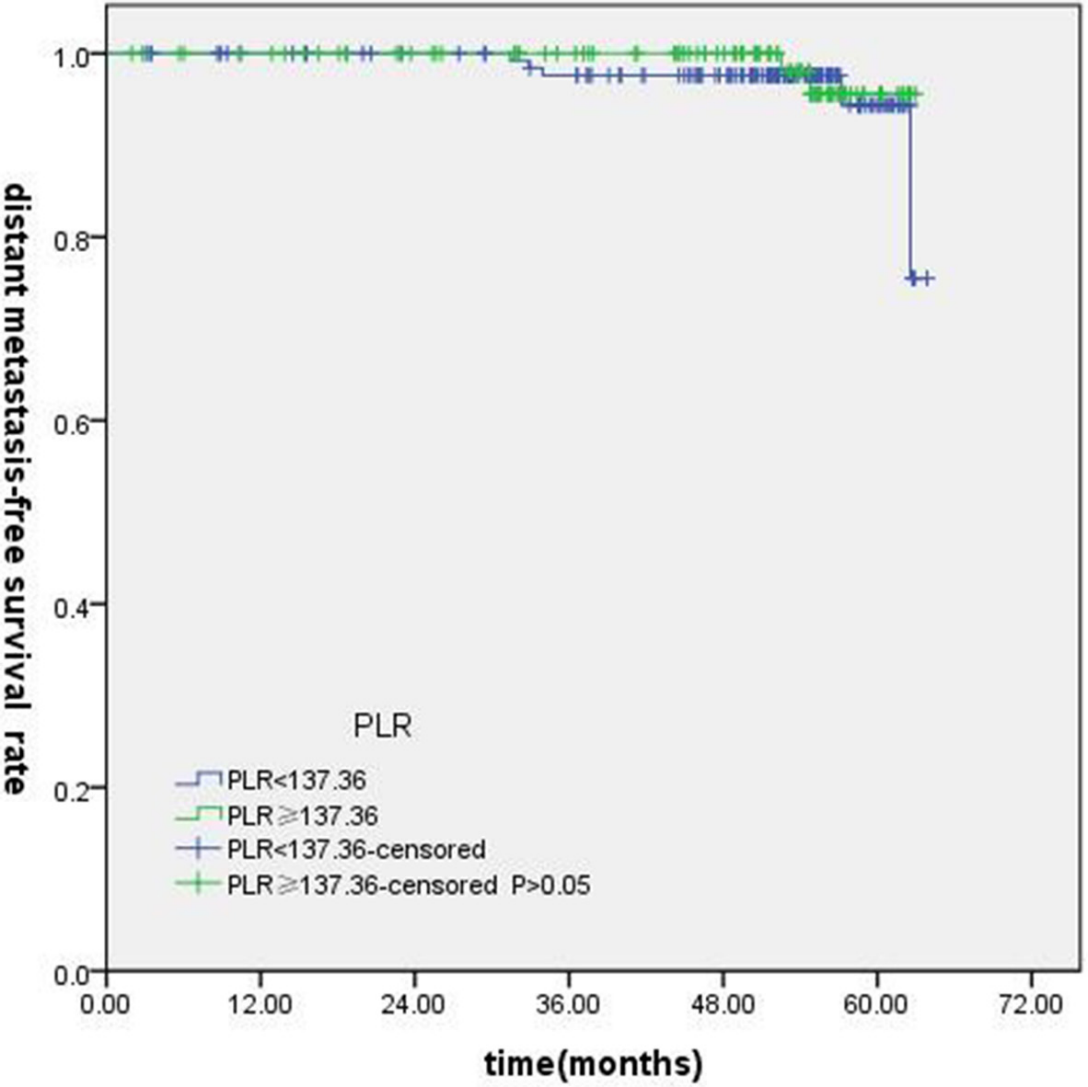
Kaplan-Merier curves for DFS according to PLR

**Figure 6:**
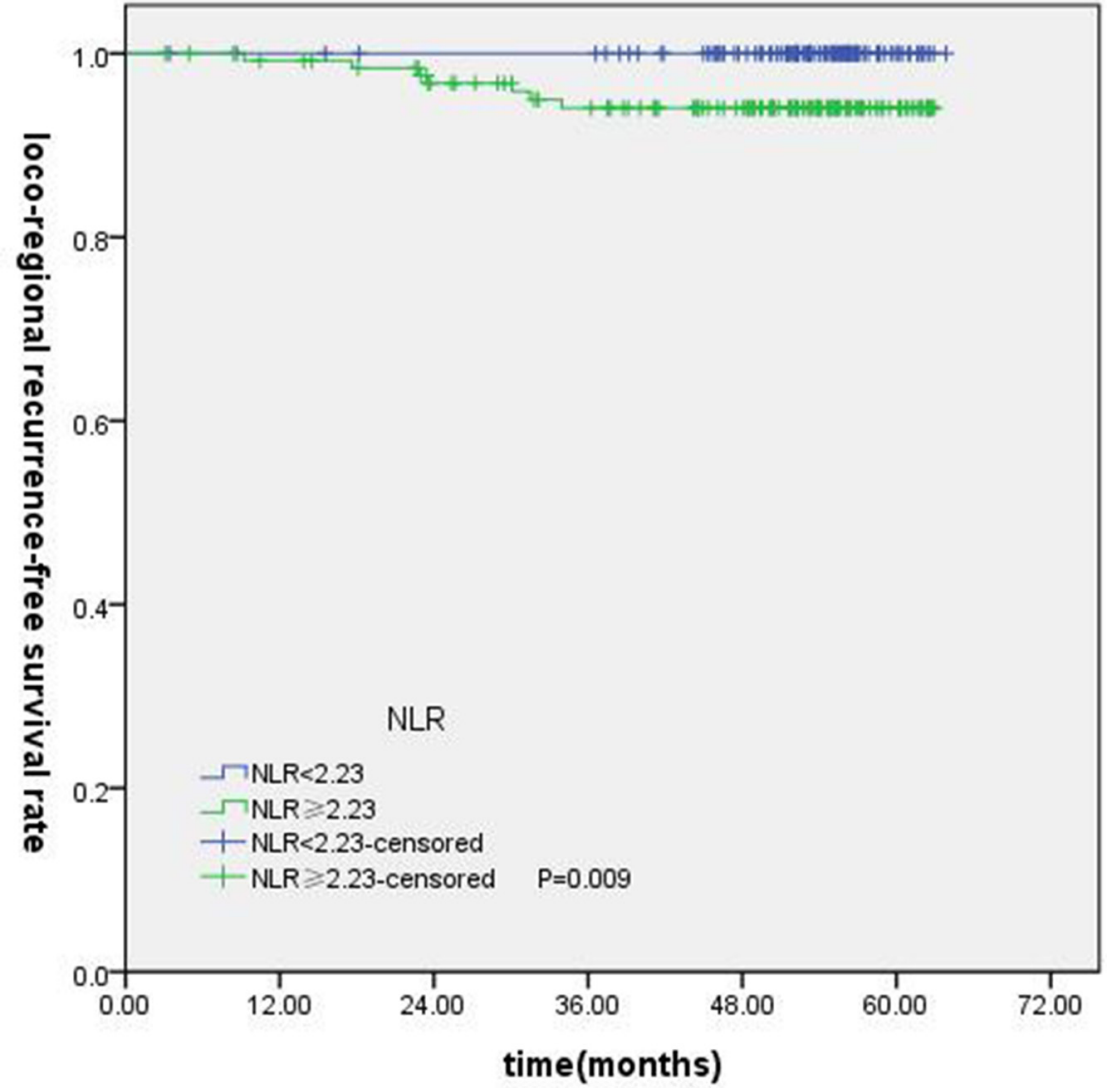
Kaplan-Merier curves for LRFS according to NLR

T-stage (*P =* 0.044), tumor stage (*P =* 0.011), NLR (*P =* 0.000) were found to be significantly associated with OS by log-rank test ( Figure 1); age (*P =* 0.004),T stage (*P =* 0.009), tumor stage (*P =* 0.002), NLR (*P =* 0.000) and PLR (*P =* 0.005) were found to be significantly associated with PFS ( Figures 2, 3); T stage (*P =* 0.009), tumor stage (*P =* 0.000), NLR (*P =* 0.000) and PLR (*P =* 0.023) were found to be significantly associated with DMFS (Figures 4, 5); NLR (*P =* 0.009) was found to be significantly associated with LRFS (Figure 6). Multivariate analysis by Cox regression showed that age (*P =* 0.000), tumor stage (*P =* 0.040), NLR (*P =* 0.004) were significantly associated with OS; age (*P =* 0.040), tumor stage (*P =* 0.038), NLR (*P =* 0.000) were indicator for PFS; tumor stage (*P =* 0.007), NLR (*P =* 0.003) were found to be significantly associated with DMFS (Table 4).

**Table 4:** Multivariable Cox proportional hazards regression analysis of OS, PFS and DMFS

Variable	OS		PFS		DMFS	
	HR(95%CI)	*P*	HR (95% CI)	*P*	HR (95% CI)	*P*
Age	4.128 (1.908–8.934)	0.000	1.851 (1.028–3.332)	0.040	N/A	N/A
T-stage	1.765 (1.391–2.494)	0.433	1.880 (1.521–2.488)	0.634	1.864 (1.466–2.603)	0.512
Tumor stage	2.926 (1.049–8.065)	0.040	2.269 (1.047–4.916)	0.038	4.361 (1.499–12.683)	0.007
NLR	3.259 (1.473–7.208)	0.004	7.093 (2.685–18.732)	0.000	6.576 (1.885–22.945)	0.003
PLR	N/A	N/A	1.242 (0.560–2.757)	0.594	0.973 (0.438–2.006)	0.868

### Subgroup analysis of NLR and PLR in locally advanced NPC

**Figure 7:**
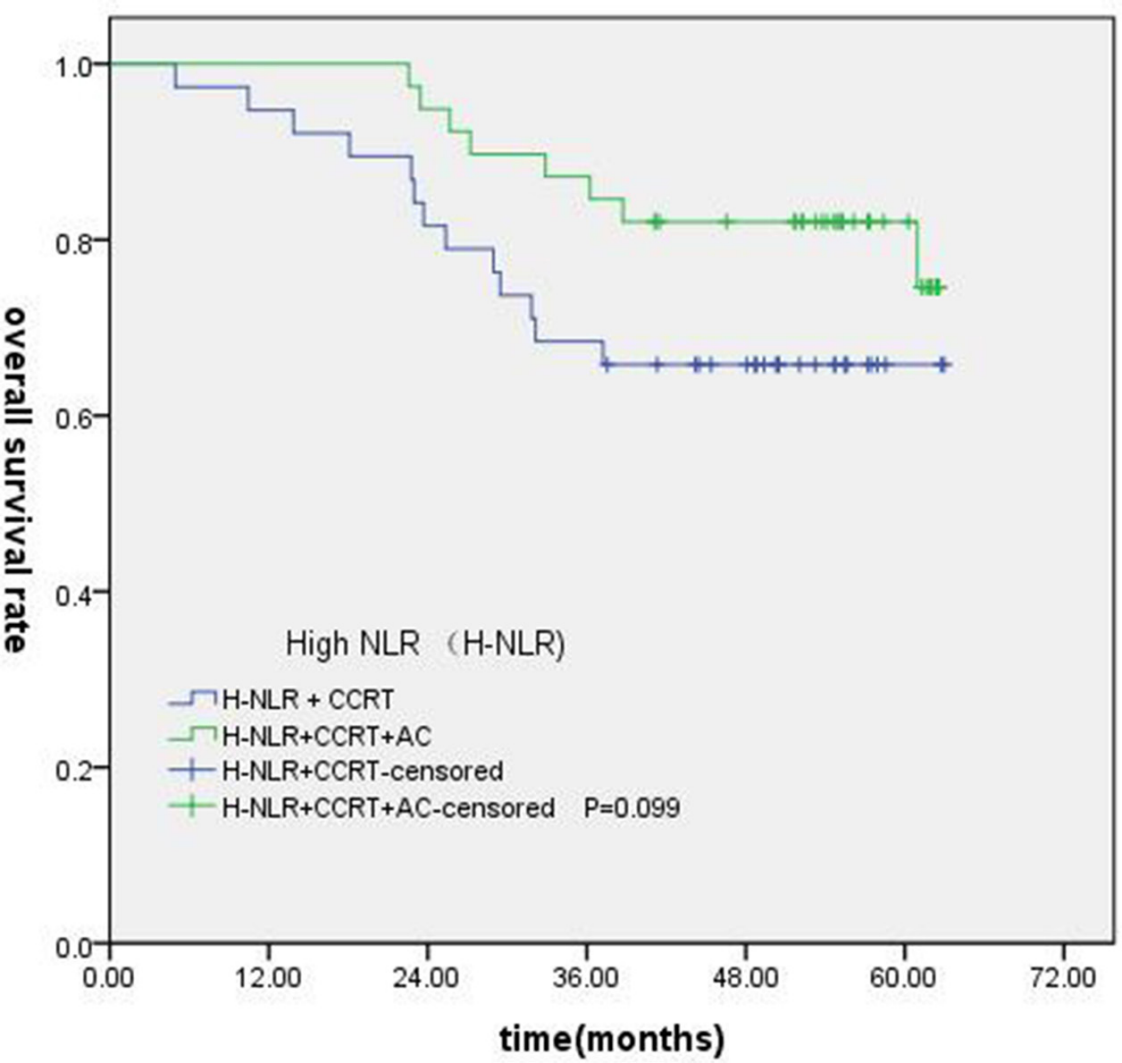
Comparison of OS of patients with locoregionally advanced NPC according to high NLR status with different treatment modalities

**Figure 8:**
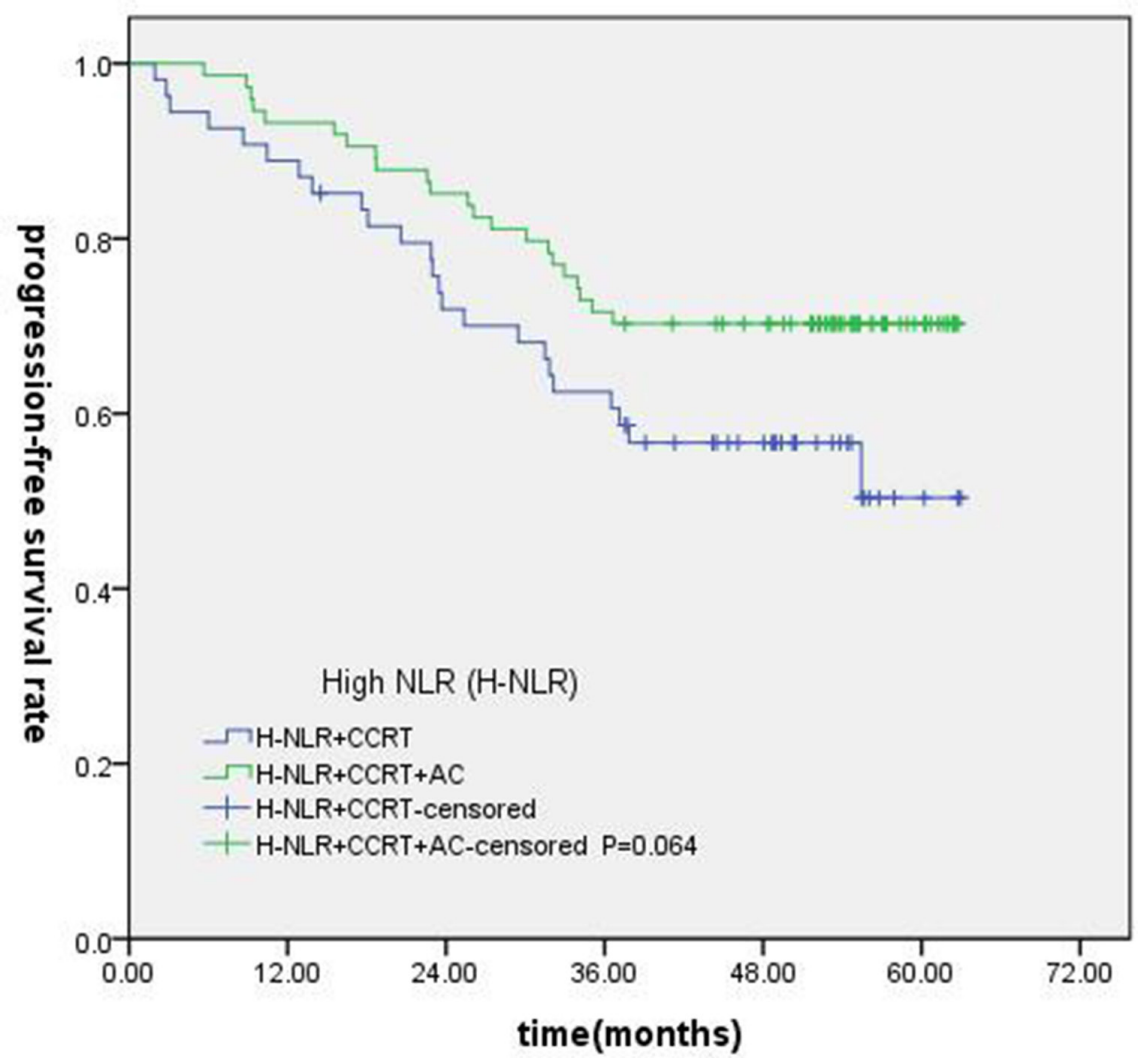
Comparison of PFS of patients with locoregionally advanced NPC according to high NLR status with different treatment modalities

**Figure 9:**
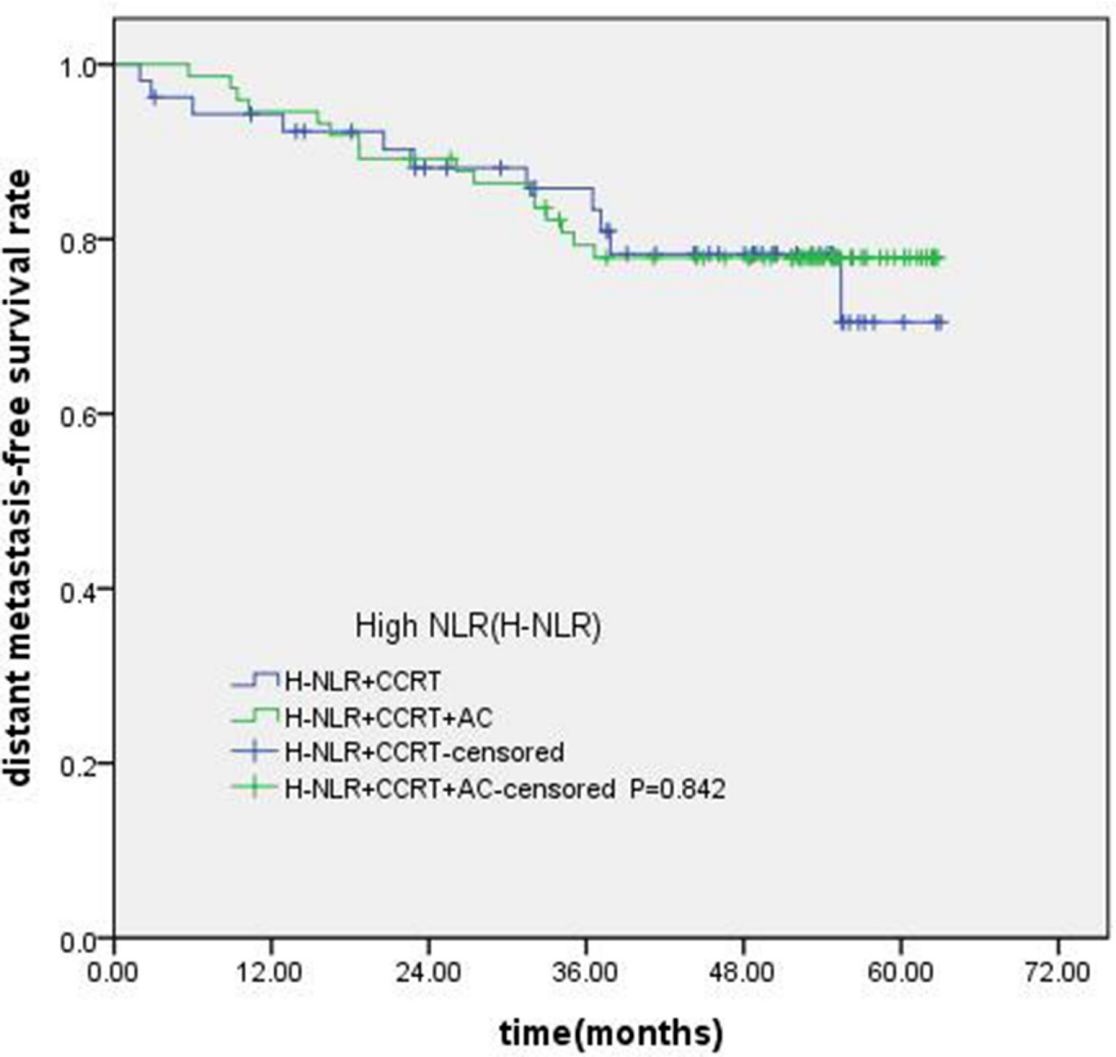
Comparison of DMFS of patients with locoregionally advanced NPC according to high NLR status with different treatment modalities

Of the 247 patients, 223 patients were locally advanced NPC. Compared with concurrent radiochemotherapy, adjuvant chemotherapy had no significantly improved OS, PFS or DMFS for patients with high NLR. (Figures 7–9).

## DISCUSSION

Since the hypothesis of Virchow, there have been more and more evidences supporting that inflammation have an impact on cancer progression [8]. The host’s immune response to tumor is lymphocyte dependent. Neutrophils is the main source of circulating angiogenesis-regulating chemokines, growth factors and proteases, which participate in tumor angiogenesis [9]. The platelets can enhance angiogenesis and release growth factors [10]. Research showed that several inflammation markers are associated with cancer, such as inflammation-based prognostic score based on C-reactive protein(CRP )and albumin levels [11, 12]. Recent reports have shown that inflammatory markers ( NLR, PLR ) can be used to predict mortality and recurrence for NPC.

In this study, we studied two markers which have reflected a systemic inflammatory response. Our result indicate that the high NLR was significantly associated with better OS, PFS and DMFS for NPC. These results are partially consistent with previous similar studies [13–15].

Several studies have confirmed that NLR and PLR were associated with the prognosis of nasopharyngeal cancer [16–25], but the results of this studies are different, which may be due to various reasons. Table 5 shows a summary of published studies on inflammatory markers in NPC. Seven researches indicated a statistically difference in the survival rates according to NLR levels regardless of the treatment method. However, only one study showed that NLR had no statistical significance for survival. This study is a comprehensive analysis of two randomized controlled trials (SQNP01 and NCC0901), found that the NLR is not a prognostic biochemical marker for locally advanced NPC. The SQNP01 is randomized trial of radiotherapy (2D-RT) versus concurrent chemoradiotherapy followed by adjuvant chemotherapy in NPC [26], and the NCC0901 is randomized trial of concurrent chemo-IMRT versus induction chemotherapy followed chemo-IMRT [27]. This two studies have treatment-specific heterogeneity, and the radiotherapy technique also be different, the addition chemotherapy could remedy the limitation of 2D-RT.

**Table 5:** Published studies of the inflammatory markers in NPC

Author(years)	No of patients	Tumor stage	radiotherapy technology	Arithmetic of cutoff value	NLR	PLR	LMR	prognosis
An X ( 2010)^19^	363	I—IVa (1997 AJCC staging system)	Unknown	ROC curve	3.73	N/A	N/A	DSS LRFS
Jian-Rong He (2012)^22^	1410	I—IV	Unknown	quartile division	1.54/1.99/2.74	N/A	N/A	OS PFS
Ying J(2013^)2^6	229	Metastatic NPC	N/A	Median value	3.6	N/A	N/A	OS
Chang H (2013)^17^	2820	I-IV (2009 AJCC staging system)	2DRT	receiver-operating characteristic analysis	2.5	300	N/A	DSS(NLR)
Li J (2013)^23^	1547	I-IV (7th edition AJCC staging system)	2DCRT/IMRT	ROC curve	N/A	N/A	5.220/4.536/4.775/5.718	OS
Chen C(2014)^24^	211	Metastatic NPC	N/A	Previously published study	5	150/300	N/A	OS(NLR)
Jiang R (2015)^18^	672	Metastatic NPC (2012 AJCC staging system)	N/A	ROC curve	N/A	N/A	2.475	OS
Sun W (2016)^25^	252	I—IV (6th edition AJCC staging system)	Unkonwn	ROC curve	2.7/2.6	167.2/163.4	N/A	OS(NLR) PFS(NLR PLR)
Chua MLK (2016)^19^	393	III-IVa,b (1997 AJCC staging system)	2DcRT/IMRT	Median value	3.0	N/A	N/A	Not a prognostic biomarker
Lu A (2017)^20^	140	I-IVa (chinese 2008 staging system)	Unkonwn	ROC curve	2.28	174	2.26	OS NLR) PFS(NLR)

N/A, Not Available;

AJCC, American Joint Committee on Cancer.

IMRT,intensity-modulated radiotherapy.

2DRT,2-dimensional radiotherapy.

LMR, lymphocyte-to-monocyte ratio.

The statistical method for determination of the cut-off value is also very important for NLR and PLR to predict prognosis. The seven researches of positive result used a fixed cutoff value regardless of the individually collected data. The cutoff values for NLR and PLR to predict prognosis has not been clearly defined in NPC. Different values were used to define the high and low NLR and PLR groups in previously published studies. Xin [20] determined the cut-off value of the NLR using ROC curve analysis, they defined a NLR > 3.73 as the high NLR group. However, Aiyin liu [19] constructed ROC curves between death events and censors, and defined the cut-off value of the NLR and PLR were 2.28 and 174 respectively. Jiang rong he [21], Melvin [18] and Ying Jin [25] used a cutoff value calculated from the quartile and median respectively. Except for the above methods, A significant majority of studies have used a cutoff value of NLR > 5, and it has been recommended that future work should use this most commonly used cutoff value in a variety of cancers [28–31]. The heterogeneity of NLR cutoff value resulted in inconsistent conclusions.

In this study, the PLR was not significantly associated with any survival outcomes on the multivariate analysis. First of all, the NLR and PLR using their respective AUC values in our study. According to the present results, the AUC for NLR were 0.720 (*P <* 0.001), 0.726 (*P <* 0.001), 0.648 (*P <* 0.001), 0.684 (*P <* 0.001) for OS, PFS, LRFS and DMFS respectively. However, the AUC for PLR were 0.579 (*P =* 0.148), 0.611(*P =* 0.013), 0.603 (*P =* 0.213), 0.624 (*P =* 0.026) for OS, PFS, LRFS and DMFS respectively. The AUCs for NLR were greater compared with PLR. Secondly, in the published studies about NPC in Table 5, only one study had shown that PLR was a prognostic indicator. The results of other similar studies in head and neck cancers had shown PLR wasn’t significantly associated with survival [32–34]. More and more studies had confirmed PLR was a prognostic factor in lung cancer and colon cancer [35, 36], but there was very few study shown that PLR related to prognosis in nasopharyngeal carcinoma, which may be caused by the tumor heterogeneity.

In our current data, we investigated the prognostic and predictive value of the NLR and PLR in 247 NPC patients without metastatic and identified the NLR as statistically significant poor prognostic factor. Similar to other results in other cancers, high NLR and PLR were significantly associated with poor prognosis. However, only NLR was an independent prognostic factor for both OS and PFS in NPC patients received IMRT.

In our study, all of the 247 patients received IMRT, 91% patients received concurrent chemotherapy, 21 (8.5%) patients added targeted drugs (cetuximab or nimotuzumab), 122 (49.4%) patients received adjuactive chemotherapy. Nonetheless, there were some heterogeneities in the treatment of NPC patients, but In the 247 included cases, 223 (90.3%) patients were III-IVa stage, our previous study had confirmed that there had no survival benefit for locally advanced NPC received addition adjuvant chemotherapy followed concurrent radiotherapy [37]. In the subgroup analysis, we found that compared with concurrent radiochemotherapy, adjuvant chemotherapy had no significantly improved survival for patients with high NLR. The standard treatment method for NPC is concurrent chemoradiotherapy, previous research [38] confirmed that concurrent chemoradiotherapy plus adjuvant chemotherapy can improved OS, Joseph also verified the similar results. Therefore, the concurrent chemoradiotherapy plus adjuvant chemotherapy became a standard method for locally advanced NPC. However, a recent meta analysis showed that addition adjuvant chemotherapy does not improve survival. [39] Most of these researches were based on conventional radiotherapy yet. However, this is a ear of IMRT, IMRT has shown remarkable benefits in local control and non-recurrence survival for patients, the role of adjuvant chemotherapy was controversial. Ladan [40] found that the value of additional adjuvant chemotherapy appears to be limited. The resaerch of chen showed concurrent chemoradiotherapy with cisplatin and fluorouracil adjuvant chemotherapy did not improve PFS in locally advanced NPC patients [41]. Resaercher had begain to pay attention to who could benefit from adjuvant chemotherapy. Zhong [42] reported concurrent chemo-IMRT with adjuvant chemotherapy might improved OS, especially for III-IVa and T4 patients. Liang [37] found that patients with N2-3 disease might benefit from the addition of adjuvant chemotherapy. In our analysis, we found that compared with concurrent radiochemotherapy, adjuvant chemotherapy had no significantly improved survival for patients with high NLR. For locally advanced NPC, NLR might not be a useful indicator for selection of treatment strategies.

Nonetheless, our research has several limitations. Firstly, being a retrospective design. Secondly, the relatively short follow-up period might limit proper prediction of long-term results. third, the small sample size might have resulted in an inadequate number of events for a proper analysis of results. Finally, The heterogeneity of the cut-off value.

## MATERIALS AND METHODS

### Patients

The data of 247 NPC patients who underwent IMRT and chemotherapy at Guangxi tumor Hospital between January 2012 and December 2012 were analyzed. The diagnosis of NPC was confirmed depended on histological evidence. Entry criteria consisted of: (1) All patients with NPC underwent IMRT with or without chemotherapy. (2) Inflammatory markers were obtained prior to anticancer treatment. (3) No hematology disease, infection, and hyperpyrexia. Finally, 247 patients were enrolled in the present study. Clinical features of eligible patients were collected including age, sex, clinical stage, dose of radiotherapy, chemotherapy, pre-treatment neutrophil count, lymphocyte count, platelet count, and NLR, PLR were calculated for each patient.

### Radiotherapy

All patients completed IMRT as planned. Patient’s head and neck were immobilized using a thermoplastic mask in the supine position. Planning CT simulation enhanced scanning of the head and neck area at 2.5 or 5 mm thickness was performed. Target volume was contoured according to the International Commission on Radiation Units and Measurements Report 50 and 62 guidelines. The gross tumor volume (GTV) and cervical lymph node tumor volume (GTVnd) were defined as the gross extent of the tumor shown by CT/MRI and physical examinations. The clinical target volume (CTV1) included the GTVnx plus 5 to 10 mm margins (forward, both sides, up and down) and a 3 to 5 mm margin (back). The CTV2 included the GTVnd, the lymphatic regions, and the CTV1 with 5 to 10 mm margins (forward, both sides, up and down) and a 3 to 5 mm margin (back). Planning target volume (PTV) was defined as CTV plus a margin of 3 mm depending on the proximity of critical structures. The radiotherapy prescription dose: PGTVnx70∼75.9 Gy/31∼32f, PGTVnd60∼73.6 Gy/30∼32f, PCTV1 60∼68 Gy/30∼31f, PCTV2 54∼57.6 Gy/30∼31f.

### Chemotherapy

223 patients received concurrent chemotherapy. Concurrent chemotherapy consist of cisplatin 100 mg/m^2^ every 3 weeks for 2–3 cycles. 121 patients received adjuvant chemotherapy. Adjuvant chemotherapy consisted of cisplatin 80 mg/m^2^ on day 1 and 5-fluorouracil 750 mg/m^2^/day by continuous intravenous infusion on 96 h every 3 weeks. 21 patients received targeted therapy. Targeted therapy contains cetuximab and nimotuzumab. An initial loading dose of cetuximab at 400 mg/m^2^ was given intravenously 1 day before IMRT, then cetuximab was given weekly at a dose of 250 mg/m^2^ for seven continuous cycles. Nimotuzumab was administered concomitantly with IMRT at a dose of 200 mg weekly for eight cycles, commenced from the first day of IMRT.

### Definition and optimal cutoff values of NLR and PLR

NLR was defined as the neutrophil counts divided by the lymphocyte counts. PLR was defined as the platelet counts divided by the lymphocyte counts. Using OS, PFS, DMFS and LRFS respectively, as end points, optimal cutoff values of NLR and PLR were obtained when the Youden index was maximal. Subsequently, patients with a NLR or PLR greater than the corresponding cutoff values were defined as high NLR or PLR, and others were defined as low NLR or PLR.

### Patient follow-up

All patients were assessed every 3 months during the first 2 years, every 6 months for the 3 subsequent years, and annually thereafter in clinic visits, telephone interviews. Physical examination, laboratory tests, and imageological diagnosis were performed at every visit. PFS was measured from the date of diagnosis to document treatment failure or last follow-up, and OS was measured from the date of diagnosis to the date of death or last follow-up, DMFS was defined as the duration from diagnosis until the date of metastasis, or date of the last follow-up. The event for LRFS was the duration between the date of being diagnosed and the date of having event of loco-regional recurrence or date of the last follow up.

### Statistical analysis

To evaluate the sensitivity and specificity for the OS, PFS, DMFS and LRFS, the receiver operating characteristic (ROC) curve was applied, and the largest Youden’s index was estimated to determine the optimal NLR and PLR cutoff values. Baseline categorical variables were summarised as frequency and percentage, and continuous variables were summarised as median with interquartile range. The chi-square test was used for comparisons of categorical datas. Comparison of continuous variables was performed using Mann-Whitney U or Kruskal-Wallis test. Survival curves were plotted by the Kaplan-Meier method, and the significance was assessed by the log-rank test. The significant predictors for OS, PFS, DMFS and LRFS determined by univariate analysis were evaluated by multivariate analysis using Cox’s proportional hazards model. All *P*-values were two-tailed and considered statistically significant if *P <* 0.05. Statistical analyses were performed using SPSS 18.0 for Windows (SPSS, Chicago, IL, USA).

## Abbreviations

NPC: Nasopharyngeal carcinoma
NLR: Neutrophil-to-lymphocyte ratio
PLR: Platelet-to-lymphocyte ratio
IMRT: Intensity-modulated radiotherapy
TNM: Tumor, Node, Metastasis
AUC: Areas under the curve
CRP: C-reactive protein
GTV: Gross tumor volume
GTVnd: Cervical lymph node tumor volume
CTV: Clinical target volume
PTV: Planning target volume
OS: Overall survival
PFS: Progression-free survival
DMFS: Distant metastasis-free survival
LRFS: Loco-regional recurrence-free survival
ROC: Receiver operating characteristic
